# Supplementary material for: Impact of secretin receptor homo-dimerization on natural ligand binding
Source: Nat Commun. 2024 May 23;15:4390. doi: 10.1038/s41467-024-48853-6 (PMC11116414; doi:10.1038/s41467-024-48853-6)
Supplement: Supplementary file 1 — Supplementary Information [file 41467_2024_48853_MOESM1_ESM.pdf]

Impact of secretin receptor homo-dimerization on natural ligand binding

Kaleeckal G. Harikumar[1], Sarah J. Piper[2,3], Arthur Christopoulos[2,3], Denise Wootten[2,3]*, Patrick M. Sexton[2,3]*, Laurence J. Miller[1]*

Supplementary Information

Supplementary Tables: 1-6

Supplementary Figures: 1-4

Supplementary Table 1: Functional characteristics of cysteine-containing SecR(G264A,I268A) constructs.

Constructs	n	pKi	B_{max} x (10^3sites/cell)	Surface expression % of SecR(G264A,I268A)
**N-terminal stalk**				
SecR (G264A,I268A)	4	8.6±0.1	33±6.6	100±0
K134C	4	9.2±0.2	25±3	60±7
R135C	4	ND	ND	71±12
H136C	4	8.9±0.2	22±6.4	73±13
S137C	4	9.3±0.2*	22±4.8	75±19
Y138C	4	ND	ND	50±8
L139C	4	ND	ND	70±9
L140C	4	8.8±0.2	30±7.5	88±15
K141C	4	ND	ND	103±16
L142C	4	ND	ND	86±18
K143C	4	ND	ND	58±9
V144C	4	9.0±0.1	29±6.9	90±9
M145C	4	ND	ND	68±4
Y146C	4	8.9±0.2	20±5.3	63±13
T147C	3	ND	ND	69±9
**ECL1**				
D196C	3	ND	ND	63±9
A197C	3	9.3±0.5	30±3.1	49±7
V198C	3	9.4±0.3	26±1.2	43±9
L199C	3	ND	ND	100±18
F200C	3	ND	ND	97±14
S201C	3	ND	ND	119±22
S202C	3	ND	ND	79±7
D203C	3	ND	ND	108±16
D204C	3	ND	ND	103±18
V205C	3	ND	ND	93±15
T206C	4	9.1±0.2	27.2±1.0	67±10
Y207C	3	ND	ND	129±18
D209C	3	9.3±0.3	30.3±4.5	79±5
A210C	3	9.1±0.1	31.5±2.6	75±9
H211C	3	ND	ND	57±9
R212C	3	8.7±0.3	77.4±22	72±13
A213C	4	9.2±0.2	43±3.8	60±11
G214C	4	9.6±0.2	37.3±3.1	91±10

K216C	5	9.1±0.1	66±7.6	62±9
L217C	3	ND	ND	98±10
V218C	4	9.7±0.3	52±5	78±14
M219C	3	ND	ND	83±19
V220C	4	9.3±0.3	79±10	77±9
L221C	3	ND	ND	92±14
F222C	3	8.7±0.5	44±7.4	73±9
Q223C	5	9.0±0.4	47±5.4	67±9
**ECL2**				
F279C	3	8.9±0.3	34±8.8	29±6
L280C	3	9.1±0.2	36±3.5	65±13
E281C	3	8.9±0.3	36±7.3	58±15
D282C	3	ND	ND	116±20
V283C	3	ND	ND	54±10
G284C	3	ND	ND	107±21
W286C	3	8.8±0.1	34.3±5.4	102±21
D287C	3	ND	ND	87±22
I288C	3	9.0±0.2	45±7.8	47±19
N289C	3	ND	ND	111±16
A290C	3	9.2±0.2	33±4.4	137±20
N291C	3	9.1±0.1	50±6.7	49±6.
A292C	3	9.0±0.2	33.7±3.3	42±6
S293C	3	9.3±0.1	41±7.1	56±6
I294C	3	9.3±0.1	36±5.1	63±8
W295C	3	8.8±0.6	43.3±3.7	71.±7
W296C	3	9.1±0.1	41.4±4.9	65±11
I297C	3	9.0±0.1	41.2±5.3	104±17
**ECL3**				
F358C	3	8.3±0.1	30.5±7.0	93±19
A359C	3	8.9±0.4	73.1±10.4	109±17
F360C	3	9.0±0.1	35.5±8.9	84±15
S361C	3	ND	ND	72±6
P362C	3	8.8±0.6	26.6±4.3	88±18
E363C	3	9.3±0.1	32.3±1.9	87±16
D364C	3	9.4±0.2	27.1±2.7	103±10
A365C	3	9.0±0.3	20±1.7	106±9
M366C	3	9.3±0.3	33.4±8.3	64±10
E367C	3	9.4±0.2	41.6±11.3	84±15
I368C	3	9.0±0.3	32.1±8.5	64±7
Q369C	3	9.1±0.3	23.1±3.9	52±6
L370C	3	9.3±0.3	41.6±10.2	95±11
F371C	3	9.2±0.3	29.9±4.1	106±13
F372C	3	8.9±0.4	44.4±7.6	62±11
E373C	3	8.5±0.3	46.1±6.9	108±12
L374C	3	8.6±0.4	68.4±15.4	65±13

Values are expressed as means ±S.E.M. from 3-5 experiments (noted above) performed in duplicate. *P<0.05, significantly different from SecR(G264A,I268A) using one-way ANOVA with Dunnett's post-test analysis. ND - no detectable binding.

Supplementary Table 2: SecR(G264A,I268A) construct cysteine-trapping studies.

Receptor constructs	Cys^{2}-Sec n=3		Cys^{5} -Sec n=3		Cys^{6}-Sec n=3		Cys^{7}-Sec n=3	
**N-terminal Stalk**	**Intraloop**	**Overall**	**Intraloop**	**Overall**	**Intraloop**	**Overall**	**Intraloop**	**Overall**
SecR (G264A,I268A)	ND	ND	ND	ND	ND	ND	ND	ND
K134C	ND	ND	ND	ND	ND	ND	ND	ND
R135C	ND	ND	ND	ND	ND	ND	ND	ND
H136C	ND	ND	ND	ND	ND	ND	ND	ND
S137C	ND	ND	ND	ND	ND	ND	ND	ND
Y138C	ND	ND	ND	ND	ND	ND	ND	ND
L139C	ND	ND	ND	ND	ND	ND	ND	ND
L140C	ND	ND	ND	ND	ND	ND	ND	ND
K141C	ND	ND	ND	ND	ND	ND	ND	ND
L142C	ND	ND	ND	ND	ND	ND	ND	ND
K143C	ND	ND	ND	ND	ND	ND	ND	ND
V144C	ND	ND	ND	ND	ND	ND	ND	ND
M145C	ND	ND	ND	ND	ND	ND	ND	ND
Y146C	ND	ND	ND	ND	ND	ND	ND	ND
T147C	ND	ND	ND	ND	ND	ND	ND	ND
**ECL1**								
D196C	ND	ND	ND	ND	ND	ND	ND	ND
A197C	ND	ND	ND	ND	ND	ND	ND	ND
V198C	ND	ND	ND	ND	ND	ND	ND	ND
L199C	ND	ND	ND	ND	ND	ND	ND	ND
F200C	ND	ND	ND	ND	ND	ND	ND	ND
S201C	ND	ND	ND	ND	ND	ND	ND	ND
S202C	ND	ND	ND	ND	ND	ND	ND	ND
D203C	ND	ND	ND	ND	ND	ND	ND	ND
D204C	ND	ND	ND	ND	ND	ND	ND	ND
V205C	ND	ND	ND	ND	ND	ND	ND	ND
T206C	ND	ND	ND	ND	ND	ND	ND	ND
Y207C	ND	ND	ND	ND	ND	ND	ND	ND
D209C	ND	ND	ND	ND	ND	ND	ND	ND
A210C	ND	ND	ND	ND	ND	ND	ND	ND
H211C	ND	ND	ND	ND	ND	ND	ND	ND
R212C	ND	ND	ND	ND	ND	ND	ND	ND
A213C	ND	ND	ND	ND	ND	ND	ND	ND
G214C	ND	ND	ND	ND	ND	ND	ND	ND
K216C	ND	ND	ND	ND	ND	ND	ND	ND
L217C	ND	ND	ND	ND	ND	ND	ND	ND
V218C	ND	ND	ND	ND	ND	ND	ND	ND
M219C	ND	ND	ND	ND	ND	ND	ND	ND
V220C	ND	ND	ND	ND	ND	ND	ND	ND
L221C	ND	ND	ND	ND	ND	ND	ND	ND
F222C	ND	ND	ND	ND	ND	ND	ND	ND
Q223C	ND	ND	ND	ND	ND	ND	ND	ND

ND - no detectable labeling above background.

Supplementary Table 3: SecR(G264A,I268A) construct cysteine-trapping studies.

Receptor constructs	**Cys^{2}-Sec** n=4		***P* values vs back ground**	**Cys^{5} -Sec** n=5		***P* values**	**Cys^{6}-Sec** n=5		***P* values**	**Cys^{7}-Sec** n=3		***P* values**
**ECL2**	**Intraloop**	**Overall**		**Intraloop**	**Overall**		**Intraloop**	**Overall**		**Intraloop**	**Overall**	
F279C	<1	<1	>0.999	<1	<1	>0.999	1.0 ±0.5	<1	>0.999	<1	<1	>0.999
L280C	<1	<1	>0.999	<1	<1	>0.999	3.2±1.9	2.6±1.9	>0.999	<1	<1	>0.999
E281C	<1	<1	>0.999	<1	<1	>0.999	3.8±2.9	3.5±3.0	>0.999	10.0 ±9.8	5.2±5.1	>0.999
D282C	<1	<1	>0.999	<1	<1	>0.999	<1	<1	>0.999	<1	<1	>0.999
V283C	<1	<1	>0.999	<1	<1	>0.999	15.8±13.9	15.5±13.9	0.9765	<1	<1	>0.999
G284C	<1	<1	>0.999	<1	<1	>0.999	1.0±0.5	<1	>0.999	<1	<1	>0.999
W286C	<1	<1	>0.999	<1	<1	>0.999	24.4±19.3	13.4±8.9	0.9941	1.2±1.1	<1	>0.999
D287C	<1	<1	>0.999	<1	<1	>0.999	4.5±3.3	4.0±3.4	>0.999	15.7±15.6	8.3±8.1	0.9915
I288C	<1	<1	>0.999	71.1±10.8	69.2±12.2*	<0.0001	4.7±3.4	4.2±3.5	>0.999	82.9±8.6	71.4±10.6*	<0.0001
N289C	<1	<1	>0.999	<1	<1	>0.999	15.8±14.5	15.7±14.5	0.9746	1.2±1.0	1.4±1.3	>0.999
A290C	<1	<1	>0.999	20.3±19.9	15.5±15.2	0.5649	1.1±0.3	1.0±0.4	>0.999	<1	<1	>0.999
N291C	<1	<1	>0.999	<1	<1	>0.999	<1	<1	>0.999	<1	<1	>0.999
A292C	<1	<1	>0.999	93.4±2.8	88.5±4.0*	<0.0001	29.1 ±17.0	24.9±17.2	0.6230	<1	<1	>0.999
S293C	<1	<1	>0.999	<1	<1	>0.999	2.6±1.8	2.5±1.8	>0.999	<1	<1	>0.999
I294C	<1	<1	>0.999	<1	<1	>0.999	10.5±9.5	10.3±9.5	0.9997	<1	<1	>0.999
W295C	100±0	93.0±7.0*	<0.0001	79.1±16.7	78.3±17.3*	<0.0001	34.2±0.1	34.1±17.2	0.2304	92.5±7.5	86.1±23.5*	<0.0001
W296C	<1	<1	>0.999	<1	<1	>0.999	80.2±19.8	68.0±20.5*	0.0003	<1	<1	>0.999
I297C	2.6±2.0	2.5±2.0	0.9990	<1	<1	>0.999	30.1±18.4	30.5±18.5	0.3563	<1	<1	>0.999
**ECL3**												
F358C	36.3±17.6	29.0±11.6	0.5256	2.2±1.8	1.8±1.4	>0.999	16.4±12.8	10.2±6.9	>0.999	21.6±20.7	21.6±20.7	0.8946
A359C	1.6±0.8	1.2±0.5	>0.999	1.4±1.0	1.2±0.7	>0.999	7.5±6.4	4.3±3.4	>0.999	85.3±8.6	85.3±8.6*	0.0004
F360C	22.9±8.3	19.4±6.5	0.9292	8.4±6.1	8.0±6.1	0.9996	8.1±6.9	4.6±3.6	>0.999	23.9±23.4	23.9±23.4	0.8213
S361C	<1	<1	>0.999	<1	<1	>0.999	3.2±2.6	2.2±1.7	0.9994	<1	<1	>0.999
P362C	7.2±5.1	7.0±5.2	>0.999	20.3±19.9	19.3±19.0	0.5279	3.5±2.2	3.2±2.3	0.999	<1	<1	>0.999
E363C	1.2±0.6	<1	>0.999	<1	<1	>0.999	2.0±1.6	2.0±1.6	0.9991	<1	<1	>0.999
D364C	22.1±19.0	21.3±19.1	0.8718	62.2±12.3	54.2±7.8*	<0.0001	17.1±8.2	16.2±8.4	>0.999	5.1±5.0	5.1±5.0	>0.999
A365C	22.5±19.5	21.7±19.6	0.8566	90.4±5.8	82.4±7.9*	<0.0001	6.1±3.9	4.7±2.7	>0.999	66.4±17.1	66.4±17.1	0.0075
M366C	2.7±2.1	1.8±1.3	>0.999	59.9±11.0	55.3±12.0*	<0.0001	56.2±16.4	48.0±17.7	0.0228	55.6±22.3	55.6±22.3	0.0354
E367C	<1	<1	>0.999	<1	<1	>0.999	40.8±17.2	35.5±14.9	0.2765	<1	<1	>0.999
I368C	<1	<1	>0.999	<1	<1	>0.999	6.4±6.0	4.3±4.0	>0.999	<1	<1	>0.999
Q369C	<1	<1	>0.999	21.6±13.4	21.6±13.5	0.3769	13.0±12.7	8.6±8.5	>0.999	<1	<1	>0.999
L370C	16.6±15.3	8.0±6.5	>0.999	<1	<1	>0.999	4.6±2.8	4.1±2.8	>0.999	1.8±1.7	1.8±1.7	>0.999
F371C	14.7±12.5	14.5±12.5	0.9943	<1	<1	>0.999	11.4±11.0	7.6±7.3	>0.999	1.8±1.7	1.8±1.7	>0.999
F372C	33.7±20.5	23.5±15.3	0.7822	<1	<1	>0.999	19.0±15.7	13.6±10.3	>0.999	24.8±24.7	24.8±24.7	0.7877
E373C	37.0±22.4	27.2±18.7	0.6098	<1	<1	>0.999	19.9±17.3	13.9±11.4	>0.999	<1	<1	>0.999
L374C	40.3±24.4	29.1±19.6	0.5199	77.3±7.7	69.8±7.5*	<0.0001	94.8±5.2	78.8±9.2*	<0.001	88.6±11.4	88.6±11.4*	0.0002

Values represent means ± S.E.M. of "n" independent experiments. Intraloop labeling represents the intensity of probe labeling each construct as a percentage of the highest labeling intensity within that loop using densitometric analysis. Overall labeling represents the intensity of probe labeling each construct as a percentage of the residue with the highest labeling intensity using that probe across all the constructs. Sites of significant labeling above background were determined using one-way ANOVA with Dunnett's post-test, with $P<0.001$ considered to be significant, and marked with *.

Supplementary Table 4: Binding characteristics of SecR constructs in membranes.

Constructs	pK_i				B_{max} picomoles/mg protein			
	Binding site 1	*P* values	Binding site 2	*P* values	Binding site1	*P* values	Binding site 2	*P* values
SecR WT	10.8±0.1		8.3±0.2		3.3±0.1		4.0±0.1	
SecR WT + GppNHp	9.2±0.2*	0.011	7.6±0.3	0.101	2.6±0.1	0.700	4.4±0.1	0.200
SecR(G264A,I268A)	9.1±0.1#	0.0136	7.1±0.1	0.100	2.7±0.1	0.700	5.5±0.1	0.100
SecR(G264A,I268A) + GppNHp	9.1±0.2	>0.999	7.6±0.3	0.400	3.1±0.1	0.700	5.1±0.1	>0.999

Values are expressed as means±S.E.M. of 3 independent experiments performed in duplicate and analyzed using Mann-Whitney test. * $P<0.05$, significantly different from control in same construct; # $P<0.05$, significantly different from the same condition at WT SecR.

Supplementary Table 5: Binding kinetics of Alexa^{488} secretin probes bound to WT and mutant SecR.

Fluorescent ligands	K_{on} x10^8 M^{-1} min^{-1}	*P* values compared to WT#	*P* values *vs* Sec-Gly^{28}-(Cys^{29}-Alexa^{488})*	K_{off} min^{-1}	*P* values compared to WT#	*P* values *vs* Sec-Gly^{28}-(Cys^{29}-Alexa^{488})*	pKi	*P* values compared to WT#	*P* values *vs* Sec-Gly^{28}-(Cys^{29}-Alexa^{488})*
**SecR WT**									
Alexa^{488}-Sec	0.9±0.4		0.754	0.7±0.1*		0.022	8.1±0.2		0.988
(Lys^{13}-Alexa^{488})Sec	0.4±0.1		0.823	0.4±0.1		0.965	7.9±0.2		0.826
(Lys^{22}-Alexa^{488})Sec	0.5±0.1		0.957	0.4±0.1		0.944	8±0.2		0.941
Sec-Gly^{28}- (Cys^{29}-Alexa^{488})	0.7±0.2			0.4±0.1			8.2±0.3		
**SecR(G264A,I268A)**									
Alexa^{488}-Sec	0.4±0.1	0.858	0.315	0.7±0.1	>0.999	0.786	7.8±0.03	0.965	0.859
(Lys^{13}-Alexa^{488})Sec	0.4±0.1	>0.999	0.251	0.8±0.1#	0.0133	0.884	7.5±0.1	0.763	0.280
(Lys^{22}-Alexa^{488})Sec	0.8±0.2	0.995	0.774	0.5±0.1	0.963	0.327	8.0±0.2	>0.999	0.997
Sec-Gly^{28}-(Cys^{29}-Alexa^{488})	1.1 ±0.5	0.886		0.7±0.4#	0.0321		7.9±0.3	0.994	

Values are expressed as means ± S.E.M. from 5 independent sets of observations performed in triplicate, and analyzed using one-way ANOVA with Tukey post-test.
*$P<0.05$, significantly different from Sec-Gly^{28}-(Cys^{29}-Alexa^{488}) ligand in the same cell lines.
#$P<0.05$, significantly different from analogous data at the WT SecR cell line.

Supplementary Table 6: Fluorescence quenching constants of secretin probes bound to WT and mutant SecR.

Probes	-GppNHp (active state)	+GppNHp (uncoupled state)	Comparison active vs uncoupled states *P* values	Comparison WT vs mutant (active state) *P* values	Comparison WT vs mutant (uncoupled state) *P* values
**SecR WT**					
$Alexa^{488}$-Sec	11.5±0.5	8.4±1.0*	0.050		
(Lys^{13}-$Alexa^{488}$)-Sec	7.9±0.6	3.9±02**	0.009		
(Lys^{22}-$Alexa^{488}$)-Sec	8.9±1.0	4.5±0.2**	0.003		
Sec-Gly^{28}-(Cys^{29}-$Alexa^{488}$)	6.2±0.7	4.9±0.4	0.805		
**SecR(G264A,I268A)**					
$Alexa^{488}$-Sec	6.3±0.5##	5.1±0.4#	0.943	0.002	0.0254
(Lys^{13}-$Alexa^{488}$)-Sec	5.2±0.4#	4.5±0.9	0.996	0.049	0.9971
(Lys^{22}-$Alexa^{488}$)-Sec	6.1±0.3#	4.3±0.2	0.263	0.048	>0.999
Sec-Gly^{28}-(Cys^{29}-$Alexa^{488}$)	5.6±1.1	4.2±0.8	0.905	0.650	>0.999

Values are expressed as means ± S.E.M. from 3 independent sets of observations analyzed using one-way ANOVA with Tukey post-test. *P<0.05, ** P<0.01, significantly different from active state of the same receptor construct; #P<0.05, ## P<0.01, significantly different from analogous condition in WT SecR.

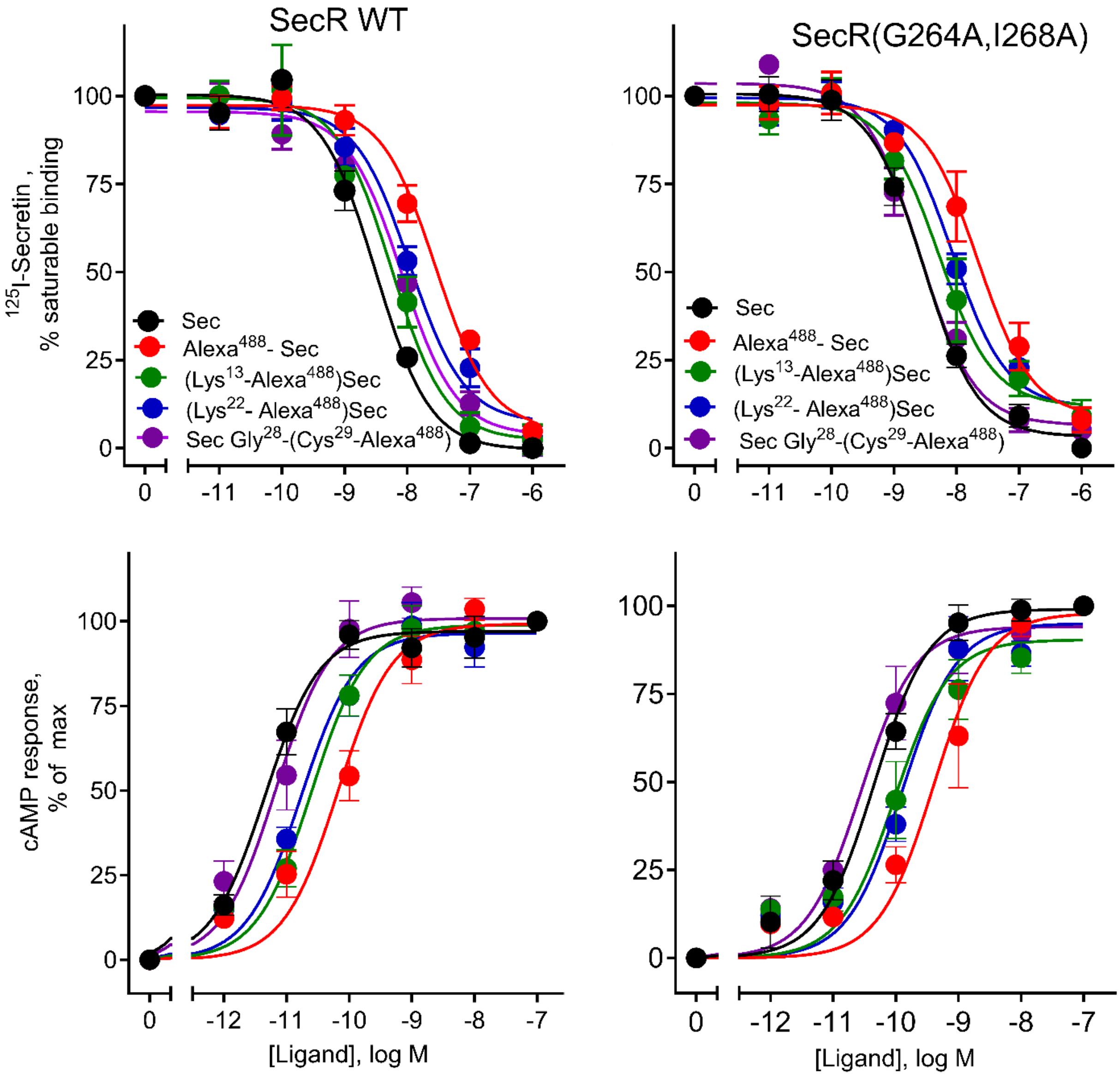

Supplementary Figure 1. Binding and biological activity characteristics of Alexa^{488}-secretin probes at cells expressing SecR constructs. Shown are curves representing the concentration-dependent competition binding (*top panel*) and cAMP responses (*bottom panel*) of fluorescent probes in cells expressing WT SecR (*left column*) and non-dimerizing mutant SecR (SecR(G264A,I268A) (*right column*). Values are expressed as percentages of saturable ^{125}I-secretin binding and percentages of maximum cAMP responses as means ± S.E.M. from 4 independent experiments performed in duplicate.

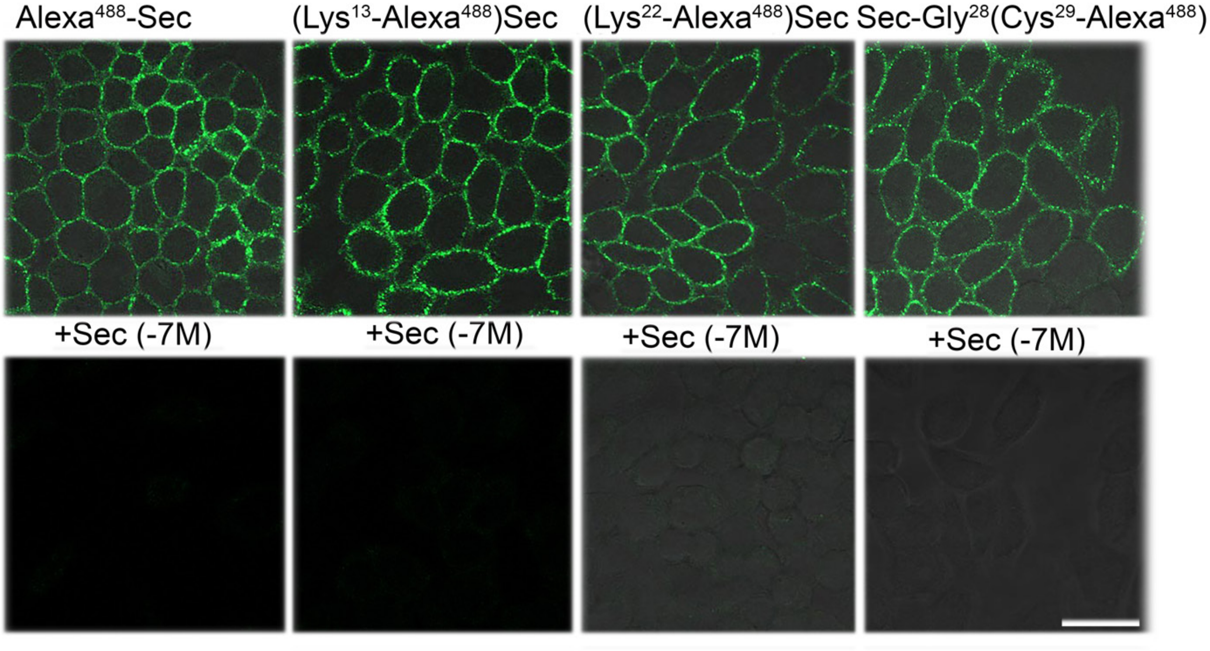

Supplementary Figure 2. Alexa^{488}-probe labeling of CHO-SecR cells. Shown are representative images illustrating surface labeling of CHO-SecR cells with Alexa^{488}-containing secretin probes. Cells were labeled with 50 nM probes for 2 h at 4°C in the absence or presence of competing 0.1 μM secretin. After incubation, the cells were washed, fixed using paraformaldehyde, washed, and imaged using confocal microscopy. Scale bar (25μm) is shown.

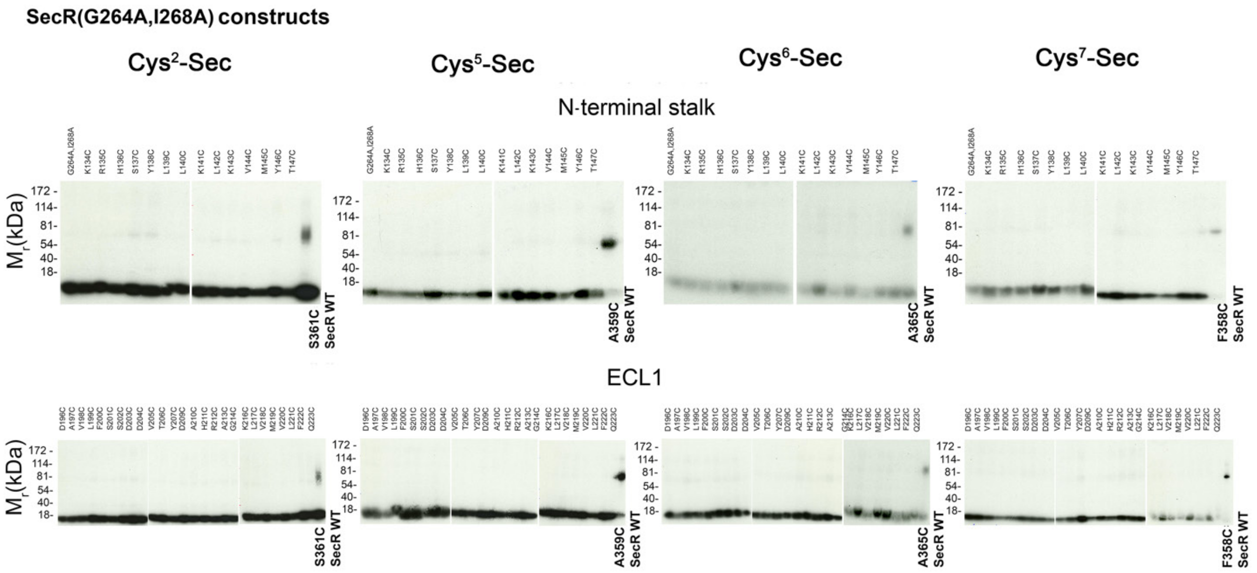

Supplementary Figure 3. Cysteine trapping of mutant SecR. Shown are representative autoradiographs of 10% SDS-PAGE gels used to separate products of cysteine trapping of SecR mutants across the N-terminal stalk and ECL1 expressed in COS-1 cells by each noted probe. Gels were run in the absence of any reducing agent, and control labeling of each region was detected from key cysteine mutants of WT SecR. Data reflect 3 independent experiments. There were no bands labeled with these probes within these regions at levels that were significantly above background.

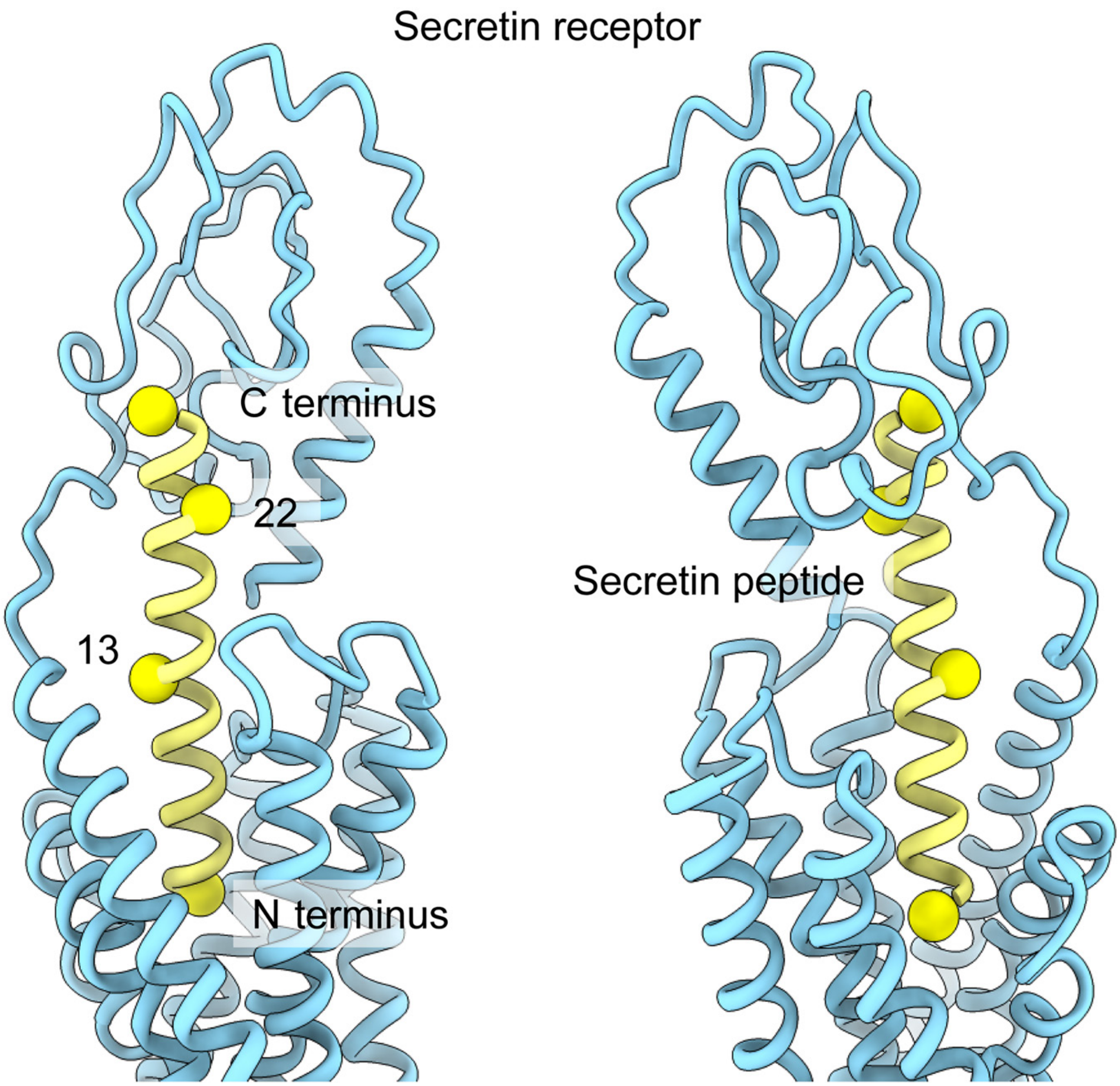

Supplementary Figure 4. Positions of fluorophores in secretin probes bound to secretin receptor. Shown are front and rear lateral views of the extracellular regions of the secretin receptor illustrating the predicted locations of fluorophores within secretin peptide probes (yellow) bound to SecR (blue), based on the published active structure (PDB: 6WZG), with positions (N terminus, 13, 22, C terminus) displayed as yellow spheres. Alexa^{488} at positions 13 and 22 are less deeply embedded in the protein than distal probes. Structure illustrations were prepared using ChimeraX version 1.6.1 (Petersen et al., Protein Sci 30., 70-82, 2021).
