# Supplementary material for: Impact of secretin receptor homo-dimerization on natural ligand binding
Source: Nat Commun. 2024 May 23;15:4390. doi: 10.1038/s41467-024-48853-6 (PMC11116414; doi:10.1038/s41467-024-48853-6)
Supplement: Supplementary file 3 — Description of Additional Supplementary Files [file 41467_2024_48853_MOESM3_ESM.pdf]

Description of Additional Supplementary Files:

Supplementary Movie 1: CryoSPARC 3D variability analysis (3DVA) with model morphs of the secretin receptor in complex with the secretin peptide and G proteins (as published previously, Dong et al., 2020). The movie shows movements of the complex in their first and second principal components from the 3DVA, focusing on the ECL2, ECL3 and peptide N terminus (first part of the movie), and the ECD and peptide C terminus (second part of the movie). Models are shown as ribbon (in grey), and maps colored by frames (frame 0, map color blue to frame 19, map color pink) of the principal component, morphing between the extreme frames, from frame 0 to frame 19 and back to frame 0. For displaying the model morphs, the backbone of the published secretin receptor structure (6WZG) was flexibly fitted into the extreme frames (frame 0 and frame 19) of each component using Isolde version 1.6 (Croll, 2018), and morphed and displayed using ChimeraX version 1.6.1 (Petersen et al, 2021). References: Dong M., Deganutti G., Piper S.J., Liang Y.L., Khoshouei M., et al. Structure and dynamics of the active Gs-coupled human secretin receptor. Nat Commun 11, 4137 (2020). Croll T.I. ISOLDE: a physically realistic environment for model building into low-resolution electrondensity maps. Acta Crystallogr D Struct Biol 74, 519-530 (2018). Pettersen E.F., Goddard T.D., Huang C.C., Meng E.C., Couch G.S., et al. UCSF ChimeraX: Structure visualization for researchers, educators, and developers. Protein Sci 30, 70-82 (2021).
